# Supplementary material for: Differences in eye movements for face recognition between Canadian and Chinese participants are not modulated by social orientation
Source: PLoS One. 2023 Dec 14;18(12):e0295256. doi: 10.1371/journal.pone.0295256 (PMC10721205; doi:10.1371/journal.pone.0295256)
Supplement: S1 File — (DOCX) [file pone.0295256.s001.docx]

**Table S1** - *Correlations: social values (AICS) and ROI (fixation percentages)*

| Learning Period | | | | |
| --- | --- | --- | --- | --- |
|  | Individualism | | Collectivism | |
| ROI | r | t | r | t |
| Eyes | -.213 | -2.283 | .213 | 2.288 |
| Center | .279 | 3.051* | -.280 | -3.059* |
| Mouth | -.002 | -0.019 | .001 | 0.006 |
| Contour | .006 | 0.067 | .013 | 0.140 |

*p < .01

| Recognition Period | | | | |
| --- | --- | --- | --- | --- |
|  | Individualism | | Collectivism | |
| ROI | r | t | r | t |
| Eyes | -.036 | -0.376 | .037 | 0.382 |
| Center | .233 | 2.502* | -.234 | -2.509* |
| Mouth | .052 | 0.548 | -.054 | -0.566 |
| Contour | .036 | 0.375 | -.035 | -0.368 |

*p < .025

*Skipped correlations (r) between ROI and AICS IND/COL dimensions for learning (n = 112) and recognition (n = 111) periods respectively. Coefficients marked with an asterisk were robust to multiple comparison correction*

**Table S2** - *Correlations: social values (AICS) and ROI (fixation percentages)*

| Learning Period | | | | |  | |  | |  | |
| --- | --- | --- | --- | --- | --- | --- | --- | --- | --- | --- |
|  | Competition | | Unique | | Responsibility | | Advice | | Harmony | |
| ROI | r | t | r | t | r | t | r | t | r | t |
| Eyes | -.460 | -5.428* | .130 | 1.377 | .200 | 2.137 | -.094 | -0.991 | .384 | 4.359* |
| Center | .111 | 1.173 | .082 | 0.867 | -.145 | -1.532 | -.109 | -1.155 | -.052 | -0.549 |
| Mouth | .063 | 0.663 | -.108 | -1.140 | -.065 | -0.681 | .108 | 1.135 | -.033 | -0.342 |
| Contour | .224 | 2.406 | -.158 | -1.675 | -.019 | -0.194 | .093 | 0.980 | -.178 | -1.901 |
| *p < .001  Recognition Period | | | | |  | |  | |  | |
|  | Competition | | Unique | | Responsibility | | Advice | | Harmony | |
| ROI | r | t | r | t | r | t | r | t | r | t |
| Eyes | -.230 | -2.469 | .169 | 1.787 | .273 | 2.967 | -.216 | -2.310 | .199 | 2.124 |
| Center | .050 | 0.524 | .135 | 1.421 | -.103 | -1.080 | -.120 | -1.264 | -.059 | -0.613 |
| Mouth | .115 | 1.204 | -.159 | -1.684 | .121 | 1.268 | .006 | 0.063 | -.122 | -1.283 |
| Contour | .286 | 3.117* | -.232 | -2.487 | -.145 | -1.526 | .080 | 0.837 | -.150 | -1.579 |

*p < .01

*Skipped correlations (r) between ROI and AICS IND/COL subdimensions for learning (n = 112) and recognition (n = 111) periods respectively. Coefficients marked with an asterisk were robust to multiple comparison correction*

**Table S3** - *Correlations: social values (HVICS) and ROI (fixation percentages)*

| Learning Period | | | | |
| --- | --- | --- | --- | --- |
|  | Individualism | | Collectivism | |
| ROI | r | t | r | t |
| Eyes | -.237 | -2.309 | .237 | 2.309 |
| Center | -.058 | -0.553 | .058 | 0.553 |
| Mouth | -.087 | -0.831 | .087 | 0.831 |
| Contour | .290 | 2.871* | -.290 | -2.871* |

*p < .01

| Recognition Period | | | | |
| --- | --- | --- | --- | --- |
|  | Individualism | | Collectivism | |
| ROI | r | t | r | t |
| Eyes | -.075 | -0.708 | .075 | 0.708 |
| Center | -.014 | -0.135 | .014 | 0.135 |
| Mouth | -.113 | -1.075 | .113 | 1.075 |
| Contour | .223 | 2.163 | -.223 | -2.163 |

*Skipped correlations (r) between ROI and HVICS IND/COL dimensions for learning (n = 92) and recognition (n = 91) periods respectively. Coefficients marked with an asterisk were robust to multiple comparison correction*

**Table S4** - *Correlations: social values (HVICS) and ROI (fixation percentages)*

| Learning Period | | | | |  | |  | |
| --- | --- | --- | --- | --- | --- | --- | --- | --- |
|  | HI | | VI | | HC | | VC | |
| ROI | r | t | r | t | r | t | r | t |
| Eyes | -.002 | 0.019 | -.379 | -3.887** | .315 | 3.147* | .031 | 0.292 |
| Center | -.107 | -1.020 | .000 | 0.003 | .007 | 0.068 | .094 | 0.898 |
| Mouth | -.001 | -0.007 | -.000 | -0.003 | .194 | 1.878 | -.141 | -1.351 |
| Contour | .095 | 0.905 | .289 | 2.864* | -.391 | -4.031** | -.034 | -0.325 |
| *p < .01  **p < .001 | | | | |  | |  | |

| Recognition Period | | | | |  | |  | |
| --- | --- | --- | --- | --- | --- | --- | --- | --- |
|  | HI | | VI | | HC | | VC | |
| ROI | r | t | r | t | r | t | r | t |
| Eyes | .223 | 2.161 | -.320 | -3.188* | .164 | 1.568 | -.114 | -1.083 |
| Center | -.037 | -0.352 | -.006 | -0.057 | .054 | 0.505 | -.052 | -0.490 |
| Mouth | .004 | 0.039 | -.046 | -0.437 | .078 | 0.741 | -.006 | 0.059 |
| Contour | -.007 | -0.063 | .296 | 2.919* | -.274 | -2.685 | -.081 | -0.767 |

*p < .01

*Skipped correlations (r) between ROI and HVICS IND/COL subdimensions for learning (n = 92) and recognition (n = 91) periods respectively. Coefficients marked with an asterisk were robust to multiple comparison correction*
